# Ensuring privacy protection in the era of big laparoscopic video data: development and validation of an inside outside discrimination algorithm (IODA)

**DOI:** 10.1007/s00464-023-10078-x

**Published:** 2023-05-05

**Authors:** A. Schulze, D. Tran, M. T. J. Daum, A. Kisilenko, L. Maier-Hein, S. Speidel, M. Distler, J. Weitz, B. P. Müller-Stich, S. Bodenstedt, M. Wagner

**Affiliations:** 1 Department for General, Visceral and Transplant Surgery, Heidelberg University Hospital, Im Neuenheimer Feld 420, 69120 Heidelberg, Germany; 2 National Center for Tumor Diseases, Heidelberg, Germany; 3 Division of Intelligent Medical Systems, German Cancer Research Center (Dkfz), Heidelberg, Germany; 4 Department for Translational Surgical Oncology, National Center for Tumor Diseases, Partner Site Dresden, Dresden, Germany; 5 Center for the Tactile Internet With Human in the Loop (CeTI), Technische Universität Dresden, Dresden, Germany; 6 Department of Visceral, Thoracic and Vascular Surgery, Faculty of Medicine and University Hospital Carl Gustav Carus, Technische Universität Dresden, Dresden, Germany; 7 Clarunis, University Center for Gastrointestinal and Liver Disease, Basel, Switzerland

**Keywords:** Artificial intelligence, Minimally invasive surgery, Prediction model, Surgical data science

## Abstract

**Background:**

Laparoscopic videos are increasingly being used for surgical artificial intelligence (AI) and big data analysis. The purpose of this study was to ensure data privacy in video recordings of laparoscopic surgery by censoring extraabdominal parts. An inside-outside-discrimination algorithm (IODA) was developed to ensure privacy protection while maximizing the remaining video data.

**Methods:**

IODAs neural network architecture was based on a pretrained AlexNet augmented with a long-short-term-memory. The data set for algorithm training and testing contained a total of 100 laparoscopic surgery videos of 23 different operations with a total video length of 207 h (124 min ± 100 min per video) resulting in 18,507,217 frames (185,965 ± 149,718 frames per video). Each video frame was tagged either as abdominal cavity, trocar, operation site, outside for cleaning, or translucent trocar. For algorithm testing, a stratified fivefold cross-validation was used.

**Results:**

The distribution of annotated classes were abdominal cavity 81.39%, trocar 1.39%, outside operation site 16.07%, outside for cleaning 1.08%, and translucent trocar 0.07%. Algorithm training on binary or all five classes showed similar excellent results for classifying outside frames with a mean F1-score of 0.96 ± 0.01 and 0.97 ± 0.01, sensitivity of 0.97 ± 0.02 and 0.0.97 ± 0.01, and a false positive rate of 0.99 ± 0.01 and 0.99 ± 0.01, respectively.

**Conclusion:**

IODA is able to discriminate between inside and outside with a high certainty. In particular, only a few outside frames are misclassified as inside and therefore at risk for privacy breach. The anonymized videos can be used for multi-centric development of surgical AI, quality management or educational purposes. In contrast to expensive commercial solutions, IODA is made open source and can be improved by the scientific community.

## Objectives

To achieve this objective, we used computer vision to develop and validate an algorithm that discriminates between inside and outside positioning of the laparoscopic camera.

## Background

In the era of big data and the ever-increasing use of artificial intelligence (AI) in surgical procedures [1], patient privacy protection is playing an increasingly important role. However, the general data protection regulation of the European Union currently represents a restriction for these possible uses [2]. Especially in the medical care system, high demands are placed on data protection [3]. Thus, surgical procedure videos, for example in laparoscopy, can still not readily be used for AI development, because outside the abdomen people could be recognizable (e.g., skin of the patient with identifying tattoo, faces of personnel). On the contrary, perfectly anonymized videos could be used and shared without the consent of the patient, because the General Data Protection Regulation does not apply to anonymous information (GDPR art. 26). More specifically, even the processing of personal data in order to fully anonymize them does not require a consent (GDPR art. 29). Similarly, anonymized data is not regulated under The Health Insurance Portability and Accountability Act (HIPAA) in the USA. Anonymized videos of the surgical procedure can be analyzed and used to ensure a high quality management and for development of decision support systems in the OR [4, 5]. Current research results show that the developed algorithms in the surgical field lack adequate data quality and especially quantity [6]. Nevertheless, patient consent is important, not only to connect surgical video data with meta-data about patient and procedure (disease stadium, blood loss, surgeon experience), but also to avoid losing the public’s trust in the scientific process.

To overcome this video shortage, we have developed and validated an inside-outside-discrimination-algorithm (IODA) that discriminates the laparoscopic camera placement inside and outside of the abdomen. As a result, IODA helps to ensure data privacy in video recordings of laparoscopic surgery by censoring extraabdominal parts that may identify patients or personnel while also maximizing the remaining video data.

There has been previous work to anonymize videos in the operating room in general. For example, Flouty et al. created a R-CNN network that detects faces in videos and consequently blurs them with a recall of up to 93.45% [7]. Disadvantage of this method is that, beside the face of a person, there might be additional security compromising details in a video. This could be the skin color, tattoos, or other identifying body morphologies. Therefore, this method is not suitable to anonymize laparoscopic videos.

By anonymizing laparoscopic videos based on inside and outside scenes, they can be safely used for big data analysis, such as surgical AI, quality management or educational purposes while immensely reducing the effort of data collection.

To realize this aim, the following research questions were investigated:

- Is it possible to reliably discriminate between inside and outside frames of laparoscopic videos using IODA based on deep neural networks?
- Does additional training information, such as classification of trocars, translucent trocars or outside for cleaning, improve algorithm performance?
- Where does the algorithm fail and does this result in privacy impairment?

## Materials and methods

### Inside outside discrimination algorithm (IODA)

**Fig. 1:**
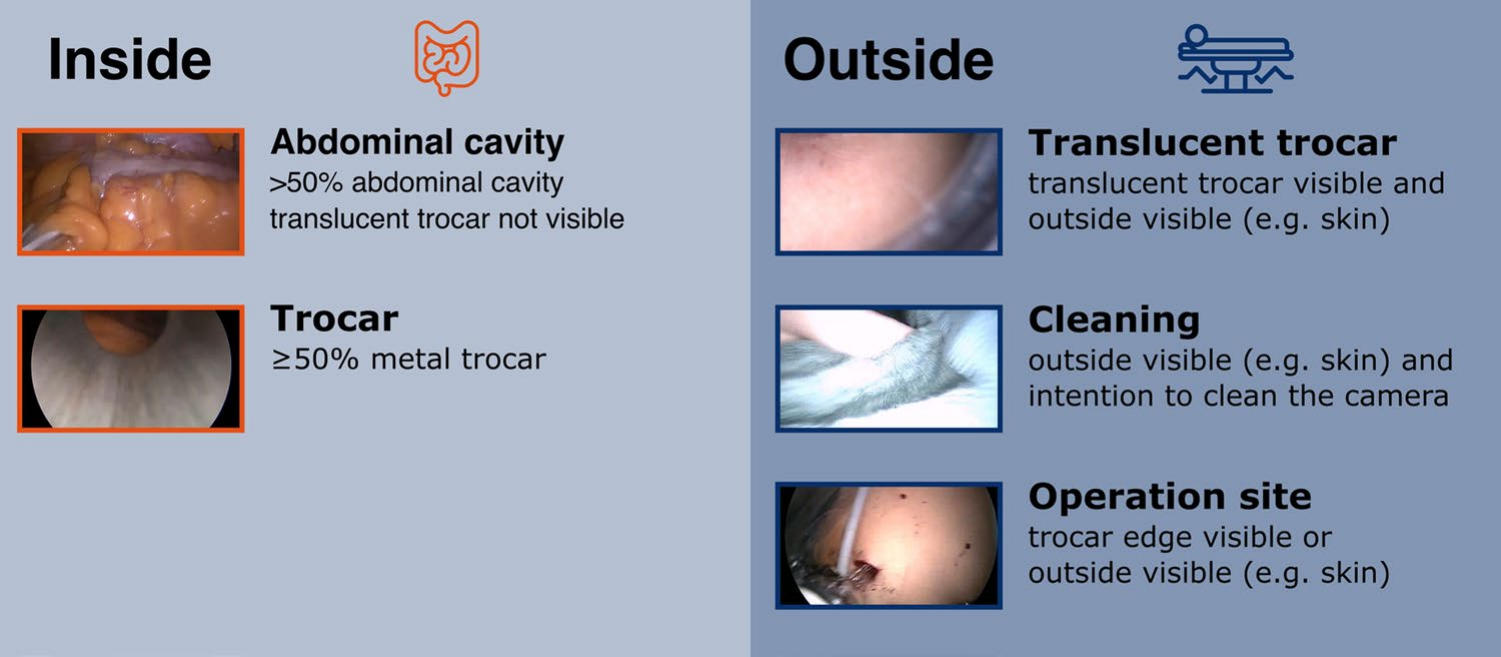
Definition of inside and outside. A frame is classified as ”Abdominal cavity” when the abdominal cavity can be seen on more than 50% of the frame and ”Trocar” when at least 50% of the frame show parts of a trocar. A frame is classified as “Translucent trocar” when any outside parts (e.g., skin) is visible through a translucent trocar. When the outside is not visible through a trocar but through the camera view directly, the frame is classified as “Operation side.” A frame is classified as “Outside for cleaning” when outside parts are visible with the intention to clean the camera

IODA has been developed to discriminate between the camera view of the inside of the abdomen and the camera view of the outside parts. Two different computer vision algorithms were developed. They share the same architecture and only differ in the number of classes they can discriminate against. One algorithm discriminates between a binary outcome (inside, outside), and the other one considers five classes (Fig. 1), two for inside (abdominal cavity, trocar) and three for outside (translucent trocar, outside, outside for cleaning).

### Deep neural network architecture

**Fig. 2:**
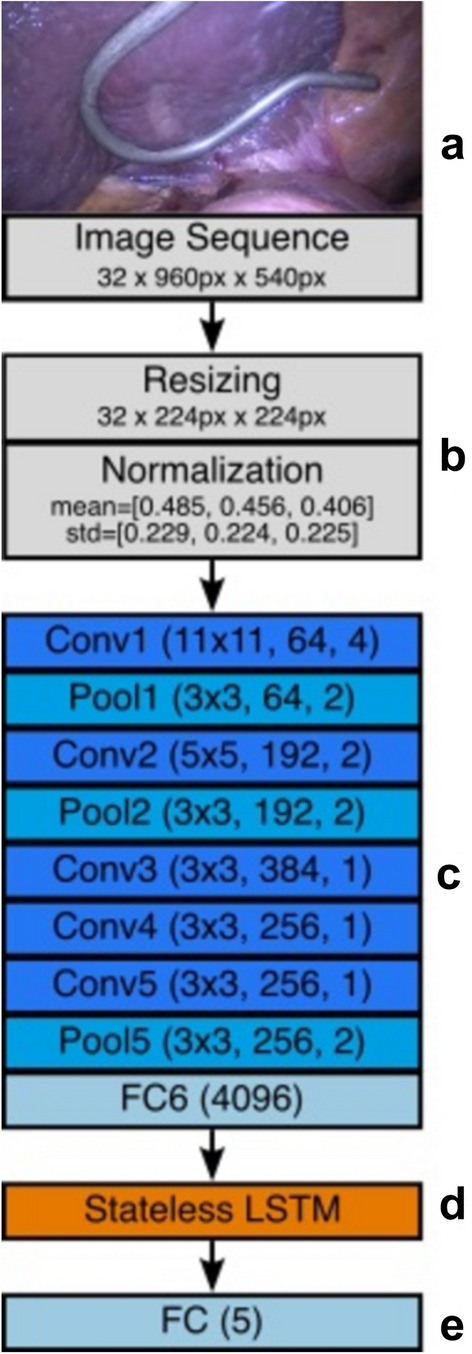
Architecture of the neural network. The neural network takes a sequence of 32 frames of resolution 960×540 as input (**a**). Each frame is resized to 224×224 and normalized with a mean of $\mu = [0.485, 0.456, 0.456]$ and standard deviation of $\sigma = [0.229, 0.224, 0.225]$ (**b**), which is the expected format for the then following AlexNet (**c**), which is pretrained on the ImageNet data set. The AlexNet is followed by a stateless long-short-term-memory (**d**) and a linear layer for classification (**e**), which returns the predicted classes for the 32 frames in the sequence. During training of the proposed neural network, transfer learning is used so that only the weights of the last linear layer need to be adjusted. *Conv* convolutional layer, *Pool* pooling layer, *FC* feature classifier, which is a linear layer, *LSTM* long-short-term-memory layer

The neural network architecture is based on the work of Bodenstedt et al. [8]. As a basis, we use the feature layers of an AlexNet and replace the classification layers by a simple dropout and linear layer with 4,096 neurons (FC6) [9], Fig. 2. The AlexNet is pretrained on the ImageNet dataset [10]. Pretraining on a diverse image set ensures that the neural net already learns to discriminate basic features like Gabor filters and color blobs [11]. These basic features are represented in the weights of the lower level hidden feature layers. This way, we can employ a technique called transfer learning, which speeds up training of the neural net by only training the upper classification layers.

Following the modified AlexNet is a stateless long-short-term-memory (LSTM). The aim of using an LSTM is that usually in a laparoscopic video each class (e.g., inside and outside) appears for an extended time and usually not as an isolated frame. Indeed, at a frame rate of 25 frames/s, an individual frame is 0.04 s long. Going from inside the abdominal cavity to outside and back in such a short time frame is very unlikely. A stateless LSTM was chosen because it analysis a single sequence of frames for correlations, but does not take into previously seen sequences. On the contrary, stateful LSTMs would memorize all sequences seen in training and are therefore more commonly used for phase recognition of laparoscopic videos, where this information is of importance [12]. Lastly, classification is done by applying a dropout of 50% and a linear layer with 2 or 5 nodes, depending on whether the binary or multiclass case was trained. The weights of this layer were adjusted during training in terms of transfer learning. As an optimization method, we opted for backpropagation using the Adam optimizer [13] due to its fast convergence and good performance when the hyperparameters are carefully chosen compared to stochastic gradient descent used in the original work for the AlexNet [14]. The training process was repeated five times on the training set, equaling 5 epochs. Additionally, mixed precision training was used for faster training speeds [15].

#### Imbalanced data

Standard metrics that are commonly used work best on balanced class distributions. In the course of annotation, it became apparent that the two classes inside and outside are not balanced. Thus, the imbalanced class distribution made an introduction of a better fitting metric necessary. We opted for the Focal Loss, which adds a modulating term to the cross-entropy to focus learning on not only imbalanced classes but specifically hard to classify classes [16]. In particular, the scaling factor decays to zero as the confidence in correctly classified frames increases.

### Experimental setup

#### Dataset

The data set contains a total of 100 laparoscopic surgery videos with 23 different operation types distributed over four categories: upper gastrointestinal, cholecystectomy, colorectal, and miscellaneous. In total, this amounts to 207 h of video (median of 1 h 30 min, [1 h 0 min, 2 h 40 min] interquartile range). Consisting of 18.6 million frames, of which only 1 frame /second (743,810 frames) was used to validate the algorithm in order to reduce computation time. The video files cover a range from short procedures, e.g., diagnostic laparoscopies, to extended procedures like laparoscopic-thoracoscopic esophagectomies performed at Heidelberg University Hospital. Table 1 gives an overview of the data set. The operation videos were recorded with a laparoscopic 2D camera (Karl Storz SE & Co KG, Tuttlingen Germany) with 30° optics, a resolution of 960 × 540 pixels and 25 frames per second. No distinction was made neither between different surgeons and their skill and experience level nor between patients and their individual case. Data analysis was approved by the local ethics committee (committee’s approval: S-248/2021).

**Table 1:** Operation types of the data set per category

Category	Operation type	Video length (mean ± standard deviation) [min]
Cholecystectomy (*n* = 19)	Cholecystectomy (*n* = 19)	55.26 ± 22.45
Upper gastrointestinal (*n* = 16)	Esophagectomy (*n* = 2)	515.00 ± 63.64
	Partial gastrectomy (*n* = 3)	83.33 ± 30.55
	Oncological total gastrectomy (*n* = 4)	345.00 ± 107.55
	Fundoplication (*n* = 6)	134.17 ± 76.71
	Sleeve gastrectomy (*n* = 1)	90.00
Colorectal (*n* = 26)	Right hemicolectomy (*n* = 6)	160.00 ± 20.82
	Left hemicolectomy (*n* = 1)	140.00
	Colectomy (*n* = 2)	160.00 ± 127.28
	Proctocolectomy (*n* = 8)	195.00 ± 46.29
	Ileocaecal resection (*n* = 3)	93.33 ± 28.87
	Sigmoid resection (*n* = 2)	140.00 ± 42.43
	Rectal resection (*n* = 4)	208.86 ± 51.56
Miscellaneous (*n* = 39)	Distal pancreatectomy (*n* = 4)	122.50 ± 29.86
	(Partial) splenectomy (*n* = 3)	86.67 ± 11.55
	Ileostomy (*n* = 2)	35.00 ± 7.07
	Intraperitoneal onlay-mesh (IPOM, *n* = 5)	83.67 ± 55.24
	Diagnostic laparoscopy (*n* = 7)	51.04 ± 36.20
	Cyst deroofing (*n* = 5)	60.00 ± 23.45
	Liver resection (*n* = 3)	125.75 ± 25.03
	Adrenalectomy (*n* = 6)	50.00 ± 8.94
	Living donor nephrectomy (*n* = 4)	212.50 ± 72.74

In our data set, a wide variety of laparoscopic procedures ensured a better generalization of the neural network

#### Data annotation

For the data annotation, the original procedure video was cut into ten minute sections and manually annotated by a medical expert using the annotation software Anvil [17]. Two main categories (inside and outside) as well as three additional sub-categories (one for inside, two for outside) were introduced (Fig. 1) While the camera view of the inside of a solid trocar can still be categorized as “Inside” (category trocar), the usage of a translucent trocar and therefore resulting in partial visibility of the patient’s skin has to be annotated as outside (category (4) translucent trocar). Another outside category is introduced to identify phases of camera being outside for cleaning the camera lens to ensure standardization and reliability of annotation, explicit rules have been defined (Fig. 1).

#### Algorithm training & testing

For algorithm training, we used a five-fold stratified cross-validation. Five equally sized sets of 20 videos were formed and alternated to form the training and test sets. To ensure that these sets are as homogeneous in total video length and operation types as possible, the original data set was manually stratified into the five sets (Table 2). Stratified splits ensure a better generalization of the neural network.

**Table 2:** Stratified fivefold cross-validation splits

Set 1	Set 2	Set 3	Set 4	Set 5
Cholecystectomy	Cholecystectomy	Cholecystectomy	Cholecystectomy	Cholecystectomy
Cholecystectomy	Cholecystectomy	Cholecystectomy	Cholecystectomy	Cholecystectomy
Cholecystectomy	Cholecystectomy	Cholecystectomy	Cholecystectomy	Cholecystectomy
Cholecystectomy	Fundoplication	Cholecystectomy	Cholecystectomy	Cholecystectomy
Esophagectomy	Fundoplication	Esophagus resection	Fundoplication	Fundoplication
Fundoplication	Gastric sleeve	Fundoplication	Oncologic total gastrectomy	Oncologic total gastrectomy
Partial gastric resection	Oncologic total gastrectomy	Oncologic total gastrectomy	Partial gastric resection	Partial gastric resection
Right hemicolectomy	Right hemicolectomy	Right hemicolectomy	Right hemicolectomy	Right hemicolectomy
Left hemicolectomy	Colectomy	Proctocolectomy	Colectomy	Right hemicolectomy
Proctocolectomy	Proctocolectomy	Proctocolectomy	Proctocolectomy	Proctocolectomy
Rectum resection	Rectum resection	Rectum resection	Sigma resection	Rectum resection
Ileocoecal resection	Ileocoecal resection	Residual proctectomy	Residual proctectomy	Ileocoecal resection
Sigma resection	Ileostoma	Splenectomy	Ileostomy	Splenectomy
Distal pancreas resection	Distal pancreas resection	Distal pancreas resection	Distal pancreas resection	Partial splenectomy
IPOM	IPOM	IPOM	IPOM	IPOM
Diagnostic laparoscopy	Diagnostic laparoscopy	Diagnostic laparoscopy	Diagnostic laparoscopy	Diagnostic laparoscopy
Cyst deroofing	Cyst deroofing	Cyst deroofing	Cyst deroofing	Cyst deroofing
Liver resection	Liver resection	Liver resection	Re-laparoscopy	Lymph node resection
Adrenalectomy	Living donor nephrectomy	Living donor nephrectomy	Living donor nephrectomy	living donor nephrectomy
Adrenalectomy	Adrenalectomy	Adrenalectomy	Adrenalectomy	Adrenalectomy

Stratified splits ensure a better generalization of the neural network.

*IPOM*  intraperitoneal onlay-mesh

IODA was trained and validated on a Gigabyte G482-Z51 gpu server (Gigabyte Technology Co. Ltd., Taipeh Taiwan) with 2 AMD 7352 CPUs (Advanced Micro Devices, Inc., Santa Clara USA) and 6 NVIDIA A40 GPUs (Nvidia Corporation, Santa Clara USA). The algorithm was written in python 3.9 [18] using the packages nvidia-dali for data loading and encoding [19], as well as, torch and torchvision for modeling the neural network [20]. There is no additional data preprocessing necessary, when running the code. This is fully integrated in the nvidia-dali pipeline which directly loads the videos and applies the necessary image transformations. The working code can be found on GitLab [https://gitlab.com/aicor/ioda].

Statistical analysis of the results is done via F1-score, sensitivity, and specificity of the outside class. The F1-score is chosen as it is the harmonic mean of precision and recall (sensitivity in the binary case) and hence a good overall metric for classification model performance. Sensitivity of the outside class indicates whether all outside frames are detected as outside, and is therefore important in terms of privacy protection. Any outside frame not detected may pose a privacy risk. Specificity of the outside class on the other hand shows how much of the inside frames are misclassified as outside. Specificity should be high to reduce the loss of valuable frames which are falsely censored.

## Results

**Fig. 3:**
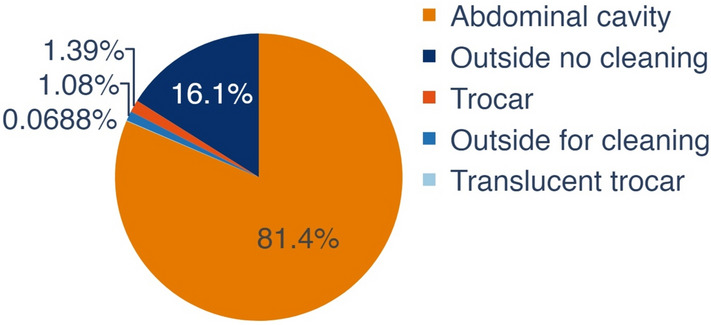
Distribution of classes. There is a class imbalance between the abdominal class (81.4%), the outside class (16.1%), and the remaining three classes (2.5%). The ground truth classes were annotated by one annotator using sequential annotation

We manually annotated 15,392,966 frames as inside and 3,202,290 frames as outside. For our multiclass experiment these frames were divided into inside abdominal cavity (15,134,978 frames, 81.39%) and trocar (257,988 frames, 1.39%) as well as outside with outside no cleaning (2,988,220 frames, 16.07%), outside for cleaning (201,282 frames, 1.08%), and translucent trocar (12,788 frames, 0.07%), as depicted by Fig. 3.

**Fig. 4:**
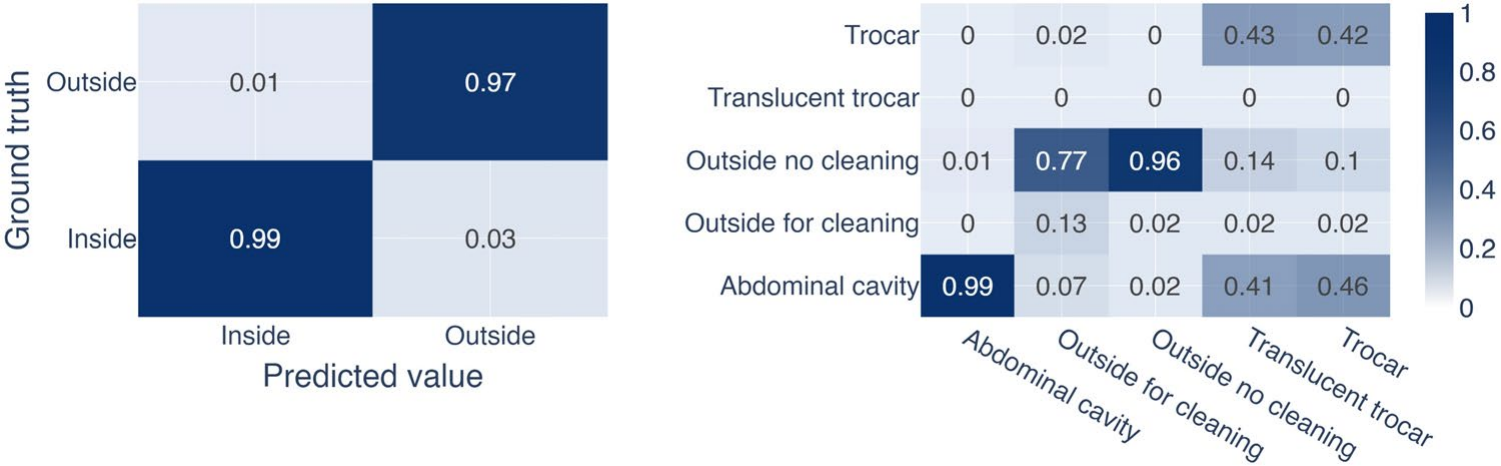
Confusion matrices for ground truth labels and predicted labels for the binary and multiclass experiment. Distribution of predicted classes for each annotated class for the binary, as well as the multiclass experiment. In the binary case, the majority class (inside) is better recognized as the minority class (outside). Similarly, for the multiclass experiment, the abdominal cavity is recognized the best by the algorithm, then outside no cleaning, which is mostly misclassified as cleaning or abdominal cavity. The trocar class is split between trocar and abdominal cavity. Frames annotated as cleaning are mostly predicted to be the operation site. The smallest class translucent trocar was never recognized by the algorithm, but instead either labeled as trocar or abdominal cavity with a similar split as the trocar class

**Fig. 5:**
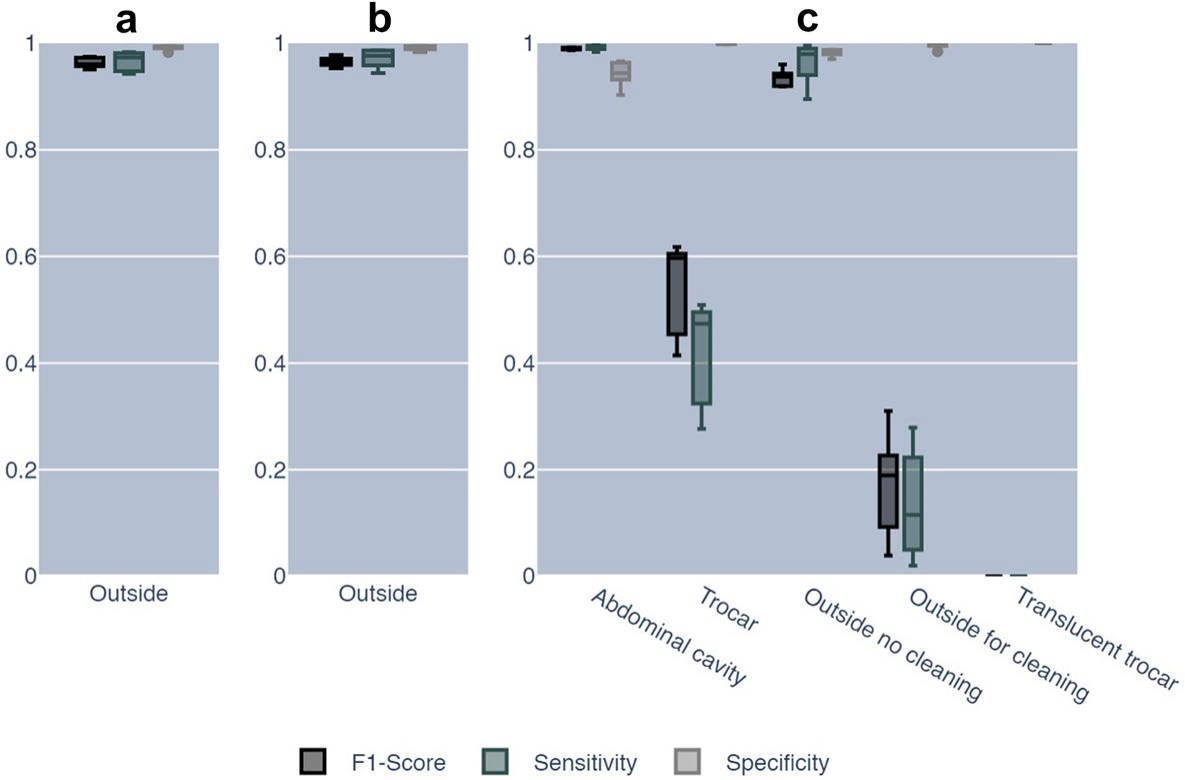
Performance of IODA. Discriminating only between inside and outside classes, the algorithm trained either on binary or multiclass labels has similarly excellent results. The video fraction which is at a security risk, due to not being recognized as outside, is quite low in both cases, as computed from the sensitivity. Similarly low is the video fraction from inside which is not recognized as inside and is consequently lost due to anonymization, as computed by the specificity. The multiclass case is additionally broken down into the individual classes. Notable is that “Cleaning,” “Translucent trocar,” and “Trocar” have a high specificity, but the algorithm is not very sensitive for these classes

When trained for binary classification, IODA matched the annotation in 611,061 out of 616,113 (99.18%) inside frames and 123,757 out of 128,079 (96.63%) outside frames (Fig. 4). Figure 5 illustrates the resulting sensitivity, specificity, and F1-score for the outside class. The sensitivity was 96.6% for “Outside” predictions, the specificity was 99.2% and the F1-Score was 0.96. A total of 9,374 frames deviate from the initial annotation, 5,052 frames are annotated as “Inside” and predicted as “Outside,” 4,322 frames are annotated as “Outside” and predicted as “Inside.”

In the multiclass experiment, the predictions for “abdominal cavity” match the annotation in 601,161 out of 605,771 (99.24%) frames. For “trocar” 10,342 frames are annotated of which are almost in equal parts predicted as “trocar” (4,345 frames, 42.01%) and “abdominal cavity” (4,773 frames, 46.15%). For “Outside for cleaning” 8,061 frames are annotated of which 1,036 (12.85%) frames are predicted as “Outside for cleaning,” while 6,242 (77.43%) frames are predicted as “Outside.” For “Outside” 119,541 frames are annotated of which 114,887 (96.10%) frames are predicted as “Outside.”

For “translucent trocar” 477 frames are annotated of which none are predicted as such, 206 (43.19%) frames are predicted as “trocar” and 196 (41.09%) frames as “abdominal cavity.”

**Fig. 6:**
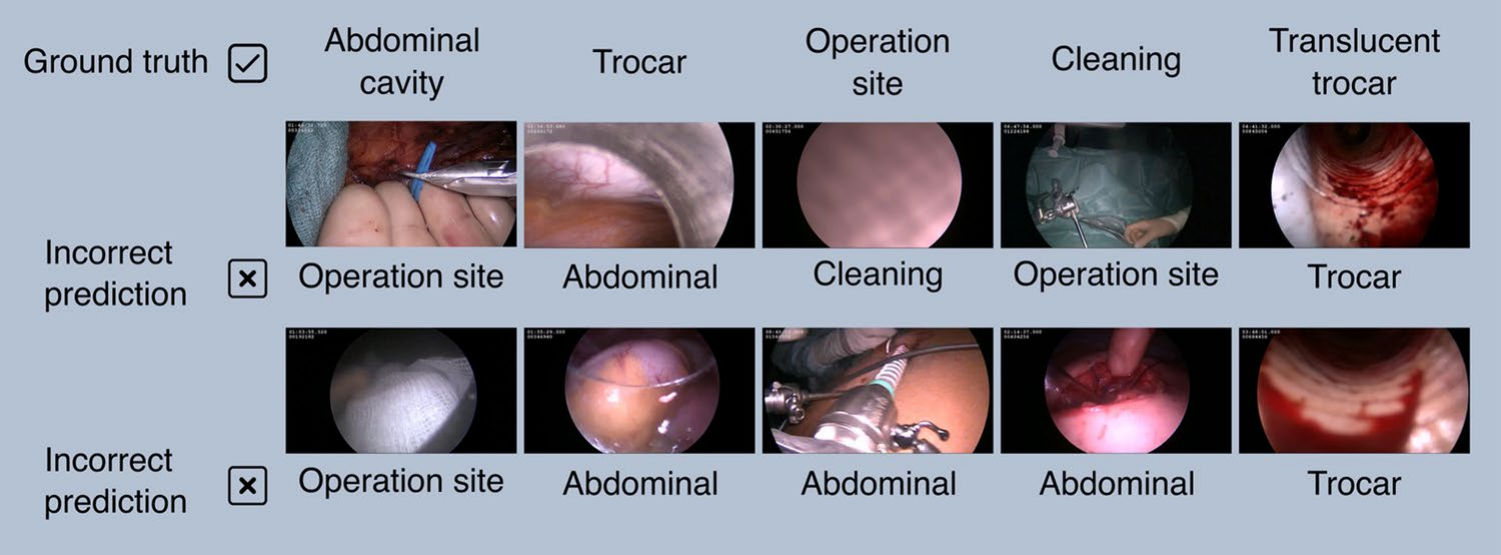
Examples of frames misclassified by IODA. The algorithm had especially problems classifying rare events and edge cases. For example, a glove appearing inside the abdominal cavity during a hand-assisted nephrectomy was classified as outside no cleaning. Misclassification of the trocar class consisted mostly of the edge cases, where the trocar and the abdominal cavity appeared to the same degree in the frame. The same is true for the translucent trocar. Additionally, the frames classified by IODA as translucent trocar were mostly trocars, which are translucent but annotated as trocar by definition because there was no skin visible in the frame. This subtle definition of classes might explain the difficulty of IODA to correctly classify the translucent trocar. Similarly, the cleaning class was defined by the intention of cleaning the camera, which can only be determined when considering quite a long sequence of frames

Out of all misclassified frames by our algorithm, there were three sequences (once 2 min and twice 15–20 s). Other than that, misclassification happened mostly in single or a series of few frames. Fig. 6 gives some examples.

## Discussion

### Data set quality

In order to provide the algorithm with a sample of laparoscopic videos as representative as possible, 23 different surgery types and a total of 100 surgeries were selected. Since the majority of a laparoscopy is situated intraabdominally, there is an inevitable class imbalance between the inside and outside classes. Regarding the outside classes, there is also high imbalance toward the class “Outside no cleaning,” which is caused by quite long sequences before and after the actual laparoscopy, where video recording was already running or was still running.

### Algorithm limitations

A striking feature of the analysis of discrepancies between manual annotation and algorithm is that the transition frames between inside and outside are especially critical and prone to errors. After analyzing the misclassified frames, especially in the multiclass experiment, it becomes apparent that the algorithm struggles with transitional areas between the camera view of the abdominal cavity and the trocar: 46.2% of frames predicted as “abdominal cavity,” 40% as “trocar.” Most probably it is caused due to our set annotation rules. The camera view of the circle and, depending on the angle, elliptical shaped trocar makes it difficult to precisely determine when it transcends 50% of the screen. Despite that all frames have been annotated to the best of the annotator's abilities, it can’t be guaranteed that the view of a category in all frames is approximated correctly. Another hurdle of the multiclass experiment is the unbalanced sampling; while frames of “abdominal cavity” make up the most part, there are only 477 used frames of a translucent trocar. The “Outside for cleaning”-category was initially intended to be used in future research projects to analyze the quantity of “Outside for cleaning”-phases and allow drawing conclusions about the complexity of the operation, the surgeon’s skill level and adapt the assisting systems. Due to the fact that this category is based on an intention, it creates a massive hurdle for our algorithm. The long-short-term memory sequences were chosen to be 32 frames, equaling 32 s of consecutive videos, which might not be long enough such that an outside sequence contains the cleaning specific activities. This is reflected in the results, where 77.4% of frames that are annotated as “Outside for cleaning” are predicted as “Outside no cleaning.”

To ensure the goal of privacy protection, it is of most importance to correctly predict outside frames as “Outside,” which is reflected by a high specificity for “Outside.” To apply our results to a practical example in case of the binary experiment: for a specificity of 99.2%, in an 1-h laparoscopic video the time span of 21 s is at risk to be falsely predicted as “Inside” while the camera view shows an outside part, while also taking into account that the video consists of 17.2% outside frames. On the other hand, the sensitivity of 96,6% results in 25 s of video data of a 1-h laparoscopic video are at risk to be lost, because it is classified as “Outside” and therefore anonymized while the camera view correctly shows the inside of the abdominal cavity. The results of the multiclass experiment show similar values as 17 s are at risk to be falsely predicted as “Inside” and 25 s are at risk to be lost.

**Fig. 7:**
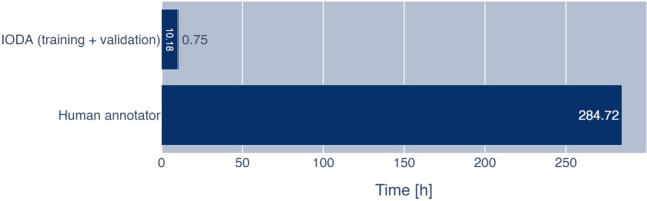
Annotation time of the human annotator and IODA. Anonymization of videos using IODA is significantly faster than a human annotator. Even when including the training time of the algorithm, using 6 NVIDIA A40, the speedup is about a factor of 26. Comparing only the validation time of IODA with the human annotator, the speedup is about 380x

While a human annotator can adapt to rare and special events, our algorithm had difficulties to classify these. Figure 7 depicts two misclassified examples for each reference annotation. As a basis, we used the multiclass experiment. For example, a misclassification occurred when a latex glove, which is almost exclusively seen outside, appeared inside the abdominal cavity during a hand-assisted living donor nephrectomy. Another common reason for failure were frames close to transitions. Frames which show approximately 50% abdominal cavity and trocar were regularly misclassified as either of the wrong classes. Fortunately, these frames are not a security risk for anonymization. Also, a cause of some misclassifications might have been the subtle definition of classes. For example, “cleaning” was defined by the intention to clean, which is very difficult to determine and usually requires a long sequence of frames. Similarly, “translucent trocar” was defined by being inside a translucent trocar and skin being visible. These sequences are usually very short and are not easy to detect, even for a human annotator. Indeed, IODA wrongly classified translucent trocars without skin being visible as the class “translucent trocar,” which by our definition belong to the “trocar” class. These examples were for the most part also not a security risk. However, some of the frames misclassified as inside contained potentially compromising information like the skin (color) of the patient.

### Advantage of algorithm over human

On the other hand, reviewing the misclassified frames showed the value and consistency of our algorithm. After checking discrepancies between IODA and the human annotator, we found obvious wrong human annotations: once because of an annotation software problem, where the annotation of a short phase had been deleted and twice because the annotator simply overlooked a short outside section. Thus, in expectation of an ever-growing database, IODA can already be expected to have an advantage and to be superior in consistency to a human annotator.

Also, noteworthy is the time needed for annotating the video, when comparing the human annotator and the computer algorithm. Figure 7 shows a comparison of the annotation time for the complete data set. Even factoring in the training time of IODA, which only has to be done once, the algorithm is significantly faster than the human annotator, approximately by a factor of 26. If we do not include the training time, this even increases to a factor of approximately 380. Obviously, the speed-up depends on the hardware, though even with less and slower graphics cards than in our setup, a real-time anonymization would be feasible.

### Potential clinical applications

**Fig. 8:**
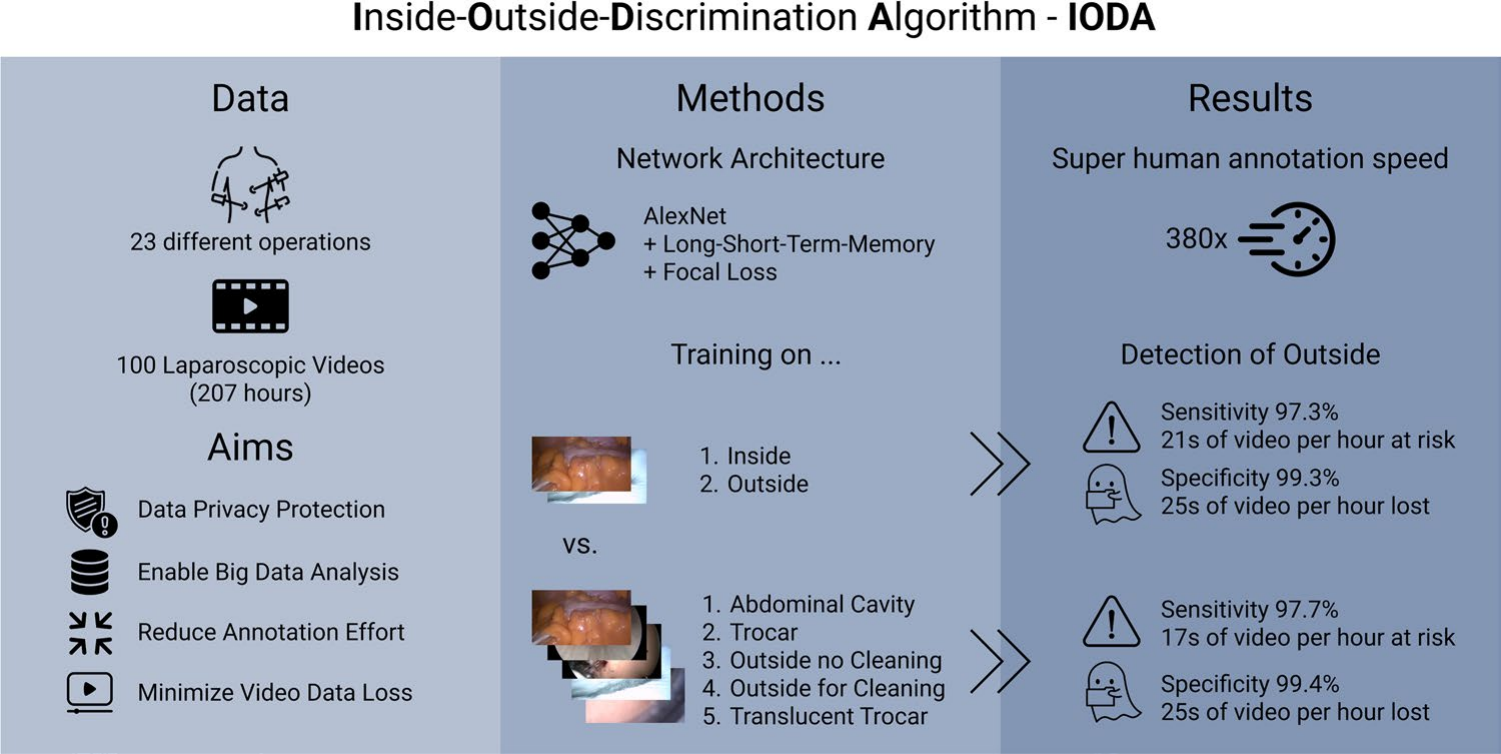
Visual abstract of IODA. The algorithm’s task is to ensure privacy protection while maximizing the remaining video data. The data set for algorithm training and testing contained a total of 100 laparoscopic surgery videos of 23 different operations. IODAs neural network architecture was based on a pretrained AlexNet augmented with a long-short-term-memory so that previous frames can influence the prediction of the following frames. False predictions were penalized by using focal loss as loss function. Algorithm training on binary or all five classes showed similarly excellent results for classifying outside frames while minimizing the lost video. The time taken to anonymize videos with IODA is significantly reduced when compared to a human annotation

In the ever-increasing digitalization, it may be possible to utilize our developed algorithm to its full potential. Due to the massively increasing video data in the clinical workspace, we would be able to build a wide and diversified database, while ensuring patients privacy protection. These anonymized videos can then be used for surgical AI development, quality management, or for educational purposes, Fig. 8. Due to the possibility of real-time application, an automated pipeline for anonymized video data would benefit other research projects in developing algorithms and in introducing AI to the broad field of surgical practice. In order to make this technology available for other surgical researchers, we made our source code, as well as the machine learning model, open source. Thus, anonymization of surgical video does not necessarily need expensive commercial solutions, but is free for the scientific community and can be refined collaboratively.

As of now, IODA runs with 45 frames per second on a hardware setup with a single NVIDIA A40 graphics card. Even if image loading and video transfer may add additional delay, the algorithm is thus suitable for “real-time” application within the operating room. This could be realized by using a medical pc with a video capture card that is connected to the video output of the laparoscope. However, the software that captures the video stream, hands it over to IODA, and displays the final video stream and potentially a graphical user interface would still need to be implemented for intraoperative real-time application.

### Future Research Directions

In future studies, the applicability of our algorithm on other operating centers with different color schemes of surgical drapes, skin and operating room surrounding, and more different operation types should be investigated. Larger data sets are essential to improve the performance of the algorithms, ideally with addition of medical device sensor data to complement manual reference annotations. Also, to speed up annotation processes, the development of time and cost-effective annotation tools should be realized. Another idea to explore that might improve the performance of IODA is a bidirectional training. Training IODA on forward and backward playing videos could increase the available data and the variability of trained scenes. Also, as explained in the methods section, the LSTM architecture of the network was chosen to take the temporal component into account, because usually each inside or outside scene is at least a couple of seconds long. In addition, a rule-based post-processing filter could be implemented that removes IODA outliers of a few frames by changing the class of very short sequences (e.g., < 2 s) to the surrounding class.

## Conclusion

Our Inside-Outside-Discrimination-Algorithm IODA allows for privacy protection when recording laparoscopic video data. Implementing this kind of deep learning into surgical video data sets holds the potential to immensely improve the quality and especially the quantity of available video data for secondary use.

The next step will be a prospective evaluation within a real time setting in the operating room.
